# KAS-Analyzer: a novel computational framework for exploring KAS-seq data

**DOI:** 10.1093/bioadv/vbad121

**Published:** 2023-09-08

**Authors:** Ruitu Lyu, Tong Wu, Gayoung Park, Yu-Ying He, Mengjie Chen, Chuan He

**Affiliations:** Department of Chemistry, The University of Chicago, IL 60637, United States; Howard Hughes Medical Institute, The University of Chicago, IL 60637, United States; Department of Chemistry, The University of Chicago, IL 60637, United States; Howard Hughes Medical Institute, The University of Chicago, IL 60637, United States; Department of Medicine, The University of Chicago, Chicago, IL 60637, United States; Department of Medicine, The University of Chicago, Chicago, IL 60637, United States; Department of Medicine, The University of Chicago, Chicago, IL 60637, United States; Department of Human Genetics, The University of Chicago, Chicago, IL 60637, United States; Department of Chemistry, The University of Chicago, IL 60637, United States; Howard Hughes Medical Institute, The University of Chicago, IL 60637, United States; Department of Biochemistry and Molecular Biology, Institute for Biophysical Dynamics, The University of Chicago, IL 60637, United States

## Abstract

**Motivation:**

Kethoxal-assisted ssDNA sequencing (KAS-seq) is rapidly gaining popularity as a robust and effective approach to study the nascent dynamics of transcriptionally engaged RNA polymerases through profiling of genome-wide single-stranded DNA (ssDNA). Its latest variant, spKAS-seq, a strand-specific version of KAS-seq, has been developed to map genome-wide R-loop structures by detecting imbalances of ssDNA on two strands. However, user-friendly, open-source computational tools tailored for KAS-seq data are still lacking.

**Results:**

Here, we introduce KAS-Analyzer, the first comprehensive computational framework aimed at streamlining and enhancing the analysis and interpretation of KAS-seq and spKAS-seq data. In addition to standard analyses, KAS-Analyzer offers many novel tools specifically designed for KAS-seq data, including, but not limited to: calculation of transcription-related metrics, identification of single-stranded transcribing (SST) enhancers, high-resolution mapping of R-loops, and differential RNA polymerase activity analysis. We provided a detailed overview of KAS-seq data and its diverse applications through the implementation of KAS-Analyzer. Using the example time-course KAS-seq datasets, we further showcase the robust capabilities of KAS-Analyzer for investigating dynamic transcriptional regulatory programs in response to UVB radiation.

**Availability and implementation:**

KAS-Analyzer is available at https://github.com/Ruitulyu/KAS-Analyzer.

## 1 Introduction

**Figure 1.**
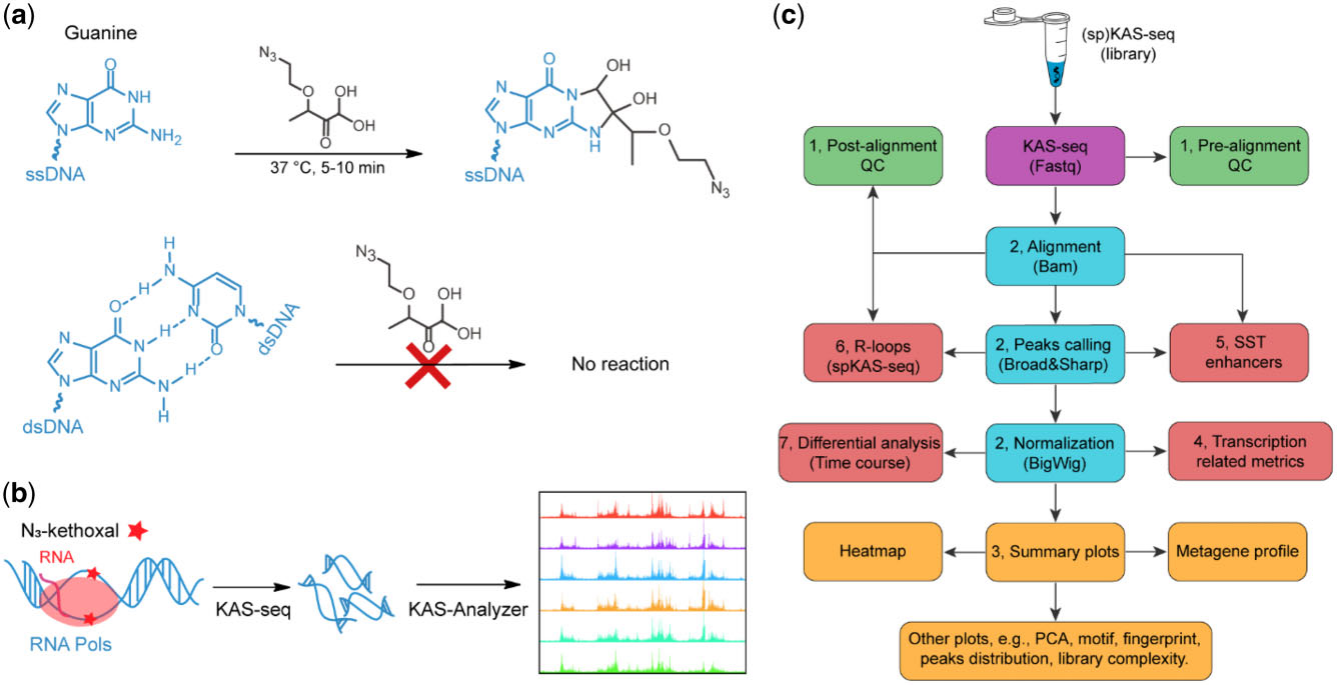
Schematic representation of KAS-seq experiments and KAS-Analyzer workflow. (a) Selectivity of N3-kethoxal for the guanine bases in ssDNA. Top: The fast chemical reaction kinetics between N3-kethoxal and exposed amine groups of guanine base. Bottom: The hydrogen bonding interactions of the Watson–Crick base pairing in dsDNA block the reaction between guanine and N3-kethoxal. (b) Schematic diagram of KAS-seq as used to map genome-wide ssDNA profiles. Red star represents the N3-kethoxal labeling. Green circle represents RNA polymerases. (c) Overview of KAS-Analyzer workflow. The colored boxes represent various analytical modules within KAS-Analyzer, with the red boxes indicating newly designed features.

Transcription, the process of synthesizing RNA molecules from a DNA template, is mediated by three different RNA polymerases (Pols) (Zhang *et al.* 2012, Chen *et al.* 2018). In eukaryotes, RNA Pol I and Pol III synthesize ribosomal RNAs (rRNAs) and transfer RNAs (tRNAs), respectively, whereas RNA Pol II mainly produces nascent messenger RNAs (mRNAs) of protein coding genes, which are subsequently processed into mature mRNAs and transported into the cytoplasm (Vannini and Cramer 2012, Wissink *et al.* 2019). Although transcriptional activities and their regulation can be traced indirectly by measuring steady-state RNA levels, only direct, real-time measurements of RNA polymerase or nascent RNA activity can reveal how transcription is dynamically regulated in response to different conditions. In recent years, many complementary approaches have been developed to detect nascent RNAs or to directly analyze the occupancy of RNA Pols, including 4SU RNA-seq, GRO-seq, PRO-seq, NET-seq, mNET-seq, and START-seq (Churchman and Weissman 2011, Min *et al.* 2011, Kwak *et al.* 2013, Fuchs *et al.* 2014, Nojima *et al.* 2015). Although these approaches use different techniques, they share common limitations, such as the necessity for millions of input cells, limited capability to detect low-abundance RNA, and susceptibility to post-transcriptional RNA processing. To overcome these challenges, we have developed kethoxal-assisted ssDNA sequencing (KAS-seq), a technology that can sensitively detect “transcription bubbles,” the short stretches of unwound DNA that form when RNA Pols engage DNA for transcription. We have shown that the transient ssDNA present in transcription bubbles provides a more direct readout of transcriptional activity *in situ* than the nascent RNAs themselves (Wu *et al.* 2020, Lyu *et al.* 2022). With the rapid (within 5 min), sensitive, and specific chemical reactions between N_3_-kethoxal and guanine bases in ssDNA, KAS-seq can work with as few as 1000 cells or even with frozen tissues (Fig. 1a and b). We have also developed a strand-specific version of KAS-seq (spKAS-seq), which enables the high-resolution mapping of R-loops by detecting asymmetric ssDNA exposure between two DNA strands (Wu *et al.* 2022).

Although the number of studies using KAS-seq has increased dramatically since its debut, existing bioinformatic methods are severely inadequate for processing KAS-seq data. Most relevant bioinformatics tools were developed for chromatin immunoprecipitation sequencing (ChIP-Seq) or chromatin accessibility data. Therefore, they cannot capture the characteristics of ssDNA profiles and will return suboptimal results when applied to KAS-seq. New computational methods specifically tailored to KAS-seq are needed to fully harness its power to survey the dynamics of transcriptionally engaged RNA Pols and other various applications. To fill the gap, we present KAS-Analyzer (Lyu 2022), the first user-friendly, flexible, and comprehensive computational framework specifically designed for the analysis of KAS-seq and spKAS-seq data. KAS-Analyzer combines state-of-the-art tools with numerous newly designed functionalities, enabling efficient processing and interpretation of KAS-seq data for a wide range of research applications. We demonstrate the capabilities of KAS-Analyzer using example datasets generated from six human cell lines (A375, HCT116, HEK293T, HeLa-S3, HepG2, and NHEK), showcasing its comprehensive workflow for investigating the dynamics of transcription regulation (Wu *et al.* 2020).

## 2 Methods

### 2.1 Installation of KAS-Analyzer

Installation instructions are provided at https://github.com/Ruitulyu/KAS-Analyzer. Before applying KAS-Analyzer on the KAS-seq data of interest, users first need to download or clone the KAS-Analyzer git repository on Github and run “bash*.*/setup.sh” to make all the scripts executable. Then, users should install all the required software with conda or mamba managers (Unix-based platforms) by executing the install tool of KAS-Analyzer. Finally, before applying the tools in KAS-Analyzer on real KAS-seq datasets, users need to activate the conda “KAS-Analyzer” environment.

### 2.2 Detailed implementations of main analytical tools in KAS-Analyzer

KAS-Analyzer was implemented using BASH scripts, R, and Python. All the tools in KAS-Analyzer toolkits can be executed under the usage: KAS-Analyzer <sub-command> [options]. The manual of KAS-Analyzer is available at https://ruitulyu.github.io/KAS-Analyzer and detailed descriptions of main analytical tools in KAS-Analyzer are included below.

### 2.3 Raw read trimming and alignment

To confidently map KAS-seq or spKAS-seq data to the reference genome of interest, KAS-Analyzer trims off low-quality sequence, adaptor sequence, and primer sequence from single-end or paired-end raw FastQ files using the trim_galore package, which can automatically detect the adaptor and primer sequences. In the “trim” tool of KAS-Analyzer, 30 bp is by default set as the shortest limit of read length after trimming off the sequence with low quality. For the read alignment of KAS-seq data, KAS-Analyzer provides two popular and top-ranked aligners, *BWA-MEM* and *Bowtie2* (Li and Durbin 2009, Langmead and Salzberg 2012). Mapped reads in sam files from the aligners are sorted and converted to bam files using “samtools sort,” which are subsequently deduplicated using “picard MarkDuplicates” or “samtools rmdup” (single-end KAS-seq data) (Li *et al.* 2009, McKenna *et al.* 2010). For single-end KAS-seq data, mapped reads were extended to 150 bp as default, regardless of the read length of raw sequencing data. For paired-end KAS-seq data, KAS-Analyzer includes a Python script that enables “properly paired” mapped reads to be combined into single interval.

### 2.4 Concurrent identification of sharp and broad KAS-seq peaks

KAS-Analyzer provides users with two popular and top-performing peak callers, *MACS2* and *epic2*, for separate sharp and broad KAS-seq peak calling. KAS-Analyzer can also integrate results from *MACS2* and *epic2* to concurrently identify sharp and broad KAS-seq peaks. By default, *MACS2* is used to call sharp peaks with a *q*-value of 0.01 and a 1.5-fold change relative to Input, while epic2 is employed to call broad peaks with a false discovery rate (FDR) of 0.05 and a 1.5-fold change compared with Input. KAS-Analyzer selects sharp peaks from *MACS2*’s results that exhibit at least a 5-fold change (versus Input) and a 2-fold change (versus shore regions) as sharp KAS-seq peaks. Broad KAS-seq peaks are defined by subtracting sharp KAS-seq peaks from peaks generated by *epic2* using *bedtools subtract*.

### 2.5 Pausing index, elongation index, and termination index calculation

KAS-Analyzer calculates the pausing index (PI) as the ratio of KAS-seq read density in promoter-proximal regions to the density in the gene bodies. Promoter-proximal regions were defined as 0.5 kb upstream and downstream of the transcription start site (TSS), and gene bodies were defined as 0.5 kb downstream of TSS to transcription end site (TES). The “bamCoverage” tool of *deeptools* is used to calculate KAS-seq read density as the number of reads per bin (50 bp), which is then normalized using Reads Per Kilobase per Million mapped reads (RPKM) (Ram**í**rez *et al.* 2014). The elongation index (EI) is calculated as the averaged KAS-seq density in the promoter-proximal and gene-body regions. The EI quantitatively measures the activity of transcribing RNA Pols. Of note, promoter-proximal and gene-body KAS-seq density are calculated independently. Similar to PI, the termination index (TI) is calculated as the ratio of KAS-seq read density at transcription–termination regions to that in the gene bodies. Transcription–termination regions were defined as 3 kb downstream of TES.

PI=ssDNA (promoter proximal)/ssDNA (gene body),

EI=average (ssDNA (promoter proximal), ssDNA (gene body)),

TI=ssDNA (termination region)/ssDNA (gene body),

where

- ssDNA(*X*) = normalized KAS-seq read density (RPKM) in region *X*,
- promoter proximal = 0.5 kb upstream and downstream of TSS,
- gene body = 0.5 kb downstream of TSS to TES,
- termination region = 3 kb downstream of TES,
- PI = pausing index,
- EI = elongation index,
- TI = termination index.

### 2.6 Single-stranded transcribing enhancers identification

Single-stranded transcribing (SST) enhancers generally denote active enhancers that overlap with KAS-seq peaks, which are mediated by proximal-paused RNA Pol II. To identify SST enhancers using KAS-seq data, KAS-Analyzer first defines enhancer shores as two regions of equal size adjacent to the upstream and downstream boundaries of the pre-defined active enhancers. Next, KAS-Analyzer calculates the ssDNA read density in each enhancer and its corresponding enhancer shores. Finally, enhancers exhibiting significantly higher ssDNA density (1.5-fold enrichment, one-way ANOVA with a *P*-value ≤ .05) compared with their enhancer shores are selected as SST enhancers.

$$\begin{array}{c} \text{SSTe}=f(E\;|\;\text{ssDNA}\left(E\right)\geq 1.5\;*\;\text{ssDNA}\left(E\;\text{shores}\right)\;\\ \text{AND}\;\text{ANOVA}\left(P-\text{value}\leq 0.05)\right), \end{array}$$

where

- SSTe = single-stranded transcribing enhancers,
- *E* = pre-defined active enhancers,
- ssDNA(*X*) = KAS-seq read density in region *X*,
- *E* shores = Enhancer shores (upstream and downstream regions adjacent to the enhancer),
- *f*(*X*) = Statistical analysis (one-way ANOVA) and fold-enrichment threshold for each pre-defined active enhancer *X*.

The list of pre-defined active enhancers can be defined by distal H3K27ac peaks or annotated by the ENCODE and Roadmap epigenomics projects (Bernstein *et al.* 2010). Moreover, KAS-Analyzer includes a “motif” tool to streamline the procedure of transcription factor motif enrichment analysis on SST enhancers using HOMER software (http://homer.ucsd.edu/homer/motif/) (Heinz *et al.* 2010).

### 2.7 Differential RNA Pols activity analysis for time-course KAS-seq data

The “TC” tool in KAS-Analyzer uses bam files of KAS-seq data at different time points as input to generate the read-count matrix. KAS-seq read count can be calculated on promoters, gene bodies, genes, KAS-seq peaks, or custom genomic bins with the “multiBigwigSummary” tool in the *deeptools* package (Ram**í**rez *et al.* 2014). The KAS-seq read-count matrix is then automatically passed to the *ImpulseDE2* package to execute the time-course KAS-seq analysis using a negative binomial noise model with dispersion trend smoothing by *DESeq2* (Love *et al.* 2014, Fischer *et al.* 2018). Finally, KAS-Analyzer creates lists of “steadily regulated genes” and “transiently regulated genes”, and produces a global heatmap plot to visualize the KAS-seq read density trajectories at different time points.

### 2.8 Genome-wide R-loop identification

R-loops can be identified genome-wide by applying KAS-Analyzer to spKAS-seq data, a function that is not available with standard KAS-seq. Unlike standard KAS-seq, spKAS-seq can be used to detect genomic regions that display an imbalance in the number of reads mapping to each DNA strand. First, the “R-loop” tool in KAS-Analyzer splits the spKAS-seq mapped reads into two groups, plus- and minus-strand mapped reads. spKAS-seq read-count matrix is then generated on the 500 bp sliding windows by default located in the *epic2* identified spKAS-seq peaks. KAS-Analyzer applied *DESeq2* to identify the sliding windows with significantly imbalanced reads among the two DNA strands using the adjusted *P*-values smaller than or equal to .05 (Love *et al.* 2014).

$$\begin{array}{c} R-\text{loops}=\{500\;\text{bp}W\;|\;\text{DESeq}2\;(\text{plus-reads}\;\left(W\right),\\ \;\;\;\;\;\;\;\;\;\;\;\;\;\;\;\text{minus-reads}\;\left(W\right),\;\text{adj}\;P\leq .05)\}, \end{array}$$

where

- R-loops = set of identified R-loop regions,
- 500 bp *W* = 500 bp sliding window located in spKAS-seq peaks,
- plus-reads (*W*) = number of spKAS-seq reads mapped to the plus strand in 500 bp window,
- minus-reads (*W*) = number of spKAS-seq reads mapped to the minus strand in 500 bp window,
- adj *P* = adjusted *P*-value from DESeq2 analysis.

The identified sliding windows were subsequently merged into combined genomic regions for R-loop definition. In addition, the “R-loop” tool also generates the R-loop density file by calculating the difference of spKAS-seq read density between two DNA strands on 50 bp bins. The “RNaseH” function in KAS-Analyzer that enables the identification of RNase H-sensitive R-loops by comparing spKAS-seq data generated from wide type and RNase H-treated cells.

R-loop density=bin | spKAS(plus-strand)-spKAS(minus-strand)|,

where

- bin = 50 bp genomic bin,
- R-loop density = absolute difference of spKAS-seq read density between two DNA strands on 50 bp bins,
- spKAS(*X*) = spKAS-seq read density on 50 bp bins in strand *X*.
  $$\begin{array}{c} \text{RNase}\;H-\text{sensitive}\;R-\text{loops}\\ =\{R-\text{loop}\;|\;R-\text{loop}\_\text{density}\_\text{compare}(R-\text{loops}\;\left(\text{wild-type}\right),\;\\ R-\text{loops}\;\left(\text{RNase}\;H-\text{treated}\right)),\;\text{FC}\leq 1.5\}, \end{array}$$

where

- RNase H-sensitive R-loops = set of identified RNase H-sensitive R-loop regions,
- R-loops(*X*) = set of R-loops identified using spKAS-seq data generated from cell type *X*,
- R-loop_density_compare(*X*, *Y*) = comparison of R-loop densities between the sets of R-loops identified in cell types *X* and *Y.*

## 3 Results

### 3.1 Overview of KAS-Analyzer workflow

KAS-Analyzer consists of eight functional modules, which can be used independently or collectively (Fig. 1c). With raw sequencing files as input, KAS-Analyzer can be used to: (1) generate pre- and post-alignment quality control metrics; (2) perform read alignment and quantification; (3) generate summary plots of KAS-seq signals in coding regions; (4) calculate transcription pausing, elongation, and TIs; (5) identify SST enhancers; (6) identify R-loops genome wide; and (7) perform differential RNA Pols activity analysis for various study designs. The computational methods for these tasks listed in modules 1, 2, and 3 are relatively well-established. KAS-Analyzer integrates existing state-of-the-art tools to execute these tasks. For instance, KAS-Analyzer employs *trim-galore* for the removal of adapter and low-quality sequences (Andrews 2010, Martin 2011), integrates *MACS2* and *epic2* for concurrent identification of sharp and broad KAS-seq peaks (Zhang *et al.* 2008, Stovner and Sætrom 2019), utilizes *idr* framework for assessing the reproducibility between replicates (Li *et al.* 2011), employs *deeptools* for metagene profiling and heatmaps, and relies on *DESeq2* or *ImpulseDE2* for differential RNA Pols activity analysis (Love *et al.* 2014, Ram**í**rez *et al.* 2014, Fischer *et al.* 2018). KAS-Analyzer has designed novel methods for the tasks outlined in modules 4, 5, 6, and 7 because no satisfactory tools have been developed up to this point. We have generated KAS-seq data from six human cell lines to offer a representative dataset and demonstrate its diverse applications. The default configuration of KAS-Analyzer serves as a best-practice toolkit and equipped with thoughtfully established parameters. In the following sections, we mainly discuss the newly designed functional modules integrated in KAS-Analyzer using the example datasets.

### 3.2 Introduction of KAS-seq data and quality control metrics

**Figure 2.**
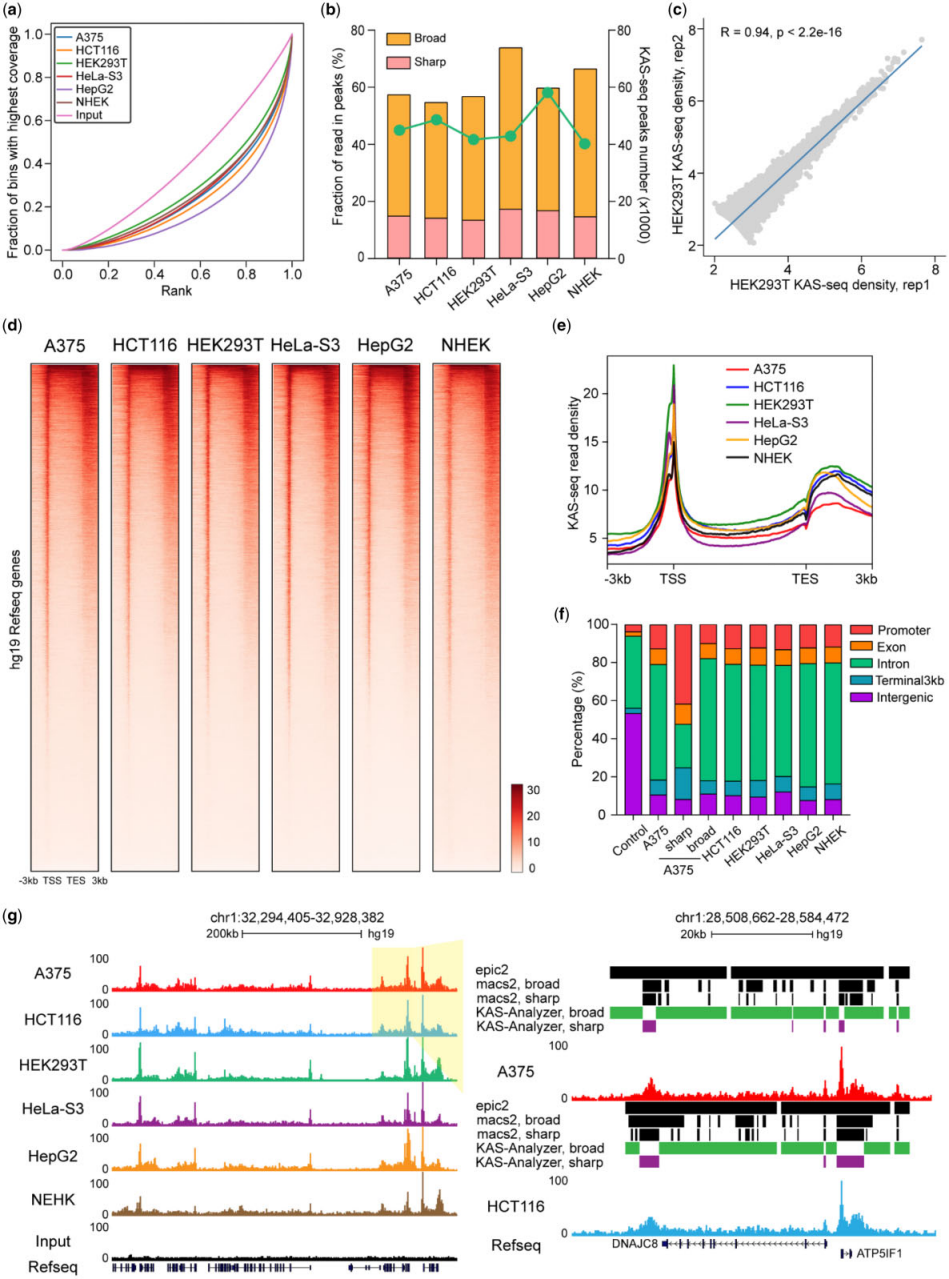
Quality control and the global view of KAS-seq data. (a) Fingerprint plot of KAS-seq data showing that KAS-seq signals can be significantly separated from the background input signals. KAS-seq data were generated in six human cell lines, including A375, HCT116, HEK293T, HeLa-S3, HepG2, and NHEK cell lines. (b) FRiP metrics of KAS-seq data showing the fraction of unique KAS-seq reads mapped on sharp and broad KAS-seq peaks in six human cell lines. The left *y*-axis represents the FRiP metric, while the right *y*-axis indicates the number of sharp and broad KAS-seq peaks. (c) Scatterplot showing the Pearson correlation between two replicates of KAS-seq data from HEK293T cells. *R*-value represents the Pearson correlation coefficient. *P*-value was determined using paired *t*-test (parametric). (d and e) Heatmap plots (d) and metagene profile (e) showing the distribution of KAS-seq read density at gene-coding regions, with 3 kb upstream of TSS and 3 kb downstream of TES shown. (f) Stacked bar plot showing the percentages of sharp and broad KAS-seq peaks distribution on different genomic features. Promoter regions were defined as 2 kb upstream and downstream of the TSS. The percentage of each genomic feature in hg19 Refseq annotation is shown as the “control.” (g) Snapshot of KAS-seq data custom tracks from UCSC Genome Browser showing the pattern of KAS-seq peaks on one example region. KAS-seq data were generated in six human cell lines. KAS-seq data in A375 and HCT116 cells highlighted in light yellow were shown on the right side of the snapshot. Sharp and broad KAS-seq peaks identified by various tools are labeled accordingly.

High-quality KAS-seq samples usually have significant ssDNA enrichment over input controls. KAS-Analyzer uses a fingerprint plot and fraction of reads in peaks (FRiP) to examine whether ssDNA signals are sufficiently enriched (Fig. 2a and b and Supplementary Fig. S1a and b). While quality control thresholds should be evaluated on a case-by-case basis, we suggest that a high-quality KAS-seq dataset should have a combined total of at least 50 000 sharp and broad KAS-seq peaks with a FRiP value greater than 40% (Fig. 2b) based on empirical KAS-seq data generated in human and mouse cell lines from our lab (Fig. 2a and b and Supplementary Fig. S1a and b). If multiple biological replicates are available, KAS-Analyzer can identify consistent peaks and generate correlation scatterplots to assess the consistency across replicates (Fig. 2c). KAS-Analyzer also includes several other tools to assess the quality of KAS-seq libraries. For instance, “KAS-Analyzer saturation” performs saturation analysis on KAS-seq data (Supplementary Fig. S1d) and “KAS-Analyzer complexity” calculates PCR bottlenecking coefficient and non-redundant fraction to assess KAS-seq library complexity (Supplementary Fig. S1e).

Previous studies show that KAS-seq exhibits a signature pattern in gene coding regions, starting with a strong and sharp peak around the TSS, followed by relatively moderate and broad signals that span the entire gene body, and ending with a strong but broad peak from the TES to its downstream region (Wu *et al.* 2020). KAS-Analyzer can examine this pattern by generating metagene profiles and heatmaps for KAS-seq signals in coding regions (Fig. 2d and e) and pie charts for the genomic distributions of KAS-seq peaks. In the example dataset, we observed that KAS-seq peaks are predominantly enriched on gene coding regions (_**∼**_90%), exhibiting enrichment in promoters (10–15%), gene bodies (60–80%), and transcription termination regions (5–10%) across each cell line (Fig. 2f). Notably, sharp KAS-seq peaks are mainly concentrated at promoters (_**∼**_50%), while broad KAS-seq peaks are primarily located within gene bodies and transcription termination regions (_**∼**_80%) (Fig. 2f and g and Supplementary Fig. S1c). Finally, KAS-Analyzer can generate KAS-seq read density files that can be viewed in the UCSC genome browser, providing users an additional option for QC through visual inspection (Fig. 2g).

### 3.3 Introduction of KAS-seq data and quality control metrics

**Figure 3.**
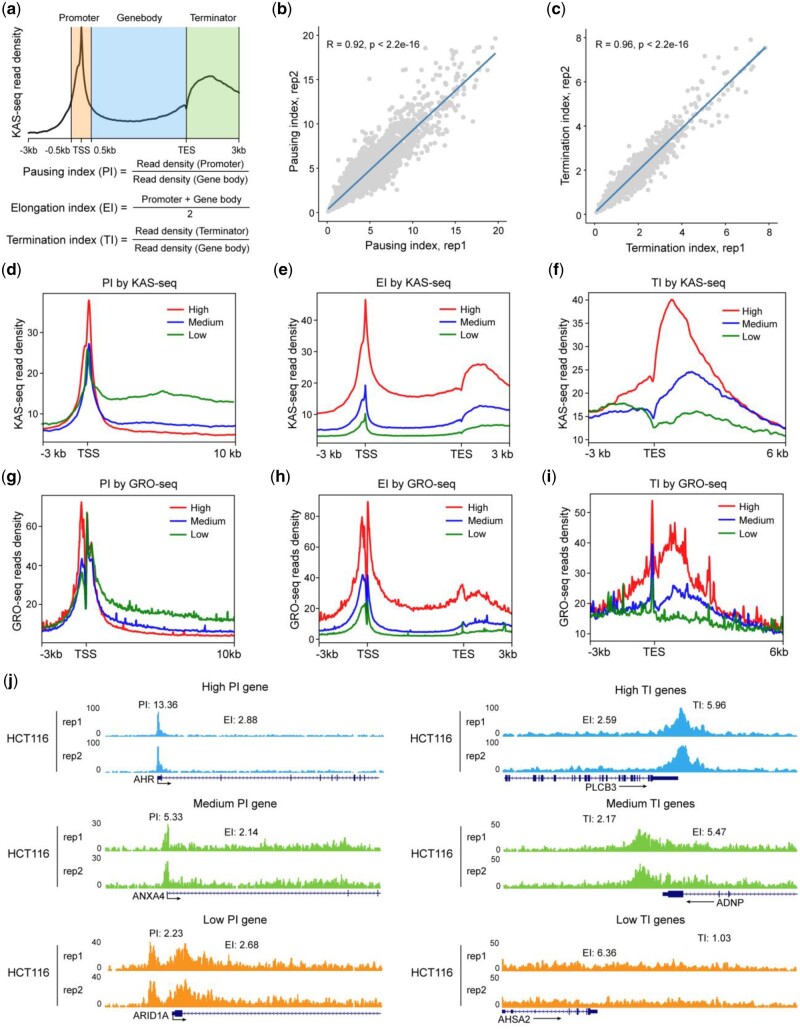
Dynamics of transcriptionally engaged RNA Pol II. (a) Schematic representation of PI, EI, and TI calculation strategy using mapped KAS-seq reads on gene coding regions. (b and c) Scatterplots showing the Pearson correlation of PI (a) and TI (b) between two biological replicates of HCT116 cells, with the *R* values denoting Pearson correlation coefficients. *P*-values were calculated using *t* distribution. (d and f) Metagene profiles showing the averaged KAS-seq read density on different groups of genes defined by PI (d), EI (e), and TI (f) metrics from HCT116 cells. Red lines represent genes with high PI, EI, and TI; blue lines represent genes with medium PI, EI, and TI; green lines represent genes with low PI, EI, and TI. (g–i) Metagene profiles showing the averaged GRO-seq read density on different groups of genes defined by PI (g), EI (h), and TI (i) metrics defined by KAS-seq data from HCT116 cells. Red lines represent genes with high PI, EI, and TI; blue lines represent genes with medium PI, EI, and TI; green lines represent genes with low PI, EI, and TI.(j) Snapshots of KAS-seq custom tracks from HCT116 cells from UCSC genome browser showing the pattern of KAS-seq reads for two replicates, rep1 (top) and rep2 (bottom), on the promoters and transcription termination regions of genes with different levels of PI (left) and TI (right). The arrows represent the transcription direction of the hg19 Refseq annotated genes.

The process of transcription can be split into three main stages: initiation, elongation, and termination (Hager *et al.* 2009). We define three metrics for protein coding genes that characterize the dynamics of transcriptionally engaged RNA Pol II: (1**)** PI, the ratio of KAS-seq read density at the promoter-proximal region (TSS+/− 500 bp) to that in the gene body (Core *et al.* 2008, Min *et al.* 2011); (2**)** EI, the averaged KAS-seq density in both the promoter-proximal region and the gene body and (3**)** TI, the ratio of KAS-seq read density in the transcription termination region to the read density in the gene body (Fig. 3a). PI is a metric originally proposed for GRO-seq data, to infer the initiation rate of paused RNA Pol II to productive elongation. EI is a newly proposed metric, which can quantitatively reflect the elongation rate of transcriptionally engaged RNA Pol II. TI is proposed to measure the efficiency of RNA Pol II “releasing” from its DNA template. To facilitate the quantification of the dynamics of transcriptionally engaged RNA Pol II, we implemented new functions in KAS-Analyzer to calculate PI, EI, and TI values for each protein-coding gene using KAS-seq data. We subsequently applied KAS-Analyzer to two replicates of HCT116 cells and found that the correlation coefficients of both PI and TI were over 0.9 between replicates, indicating that the calculation of these indexes is robust and reproducible (Fig. 3b and c). Additionally, we plotted GRO-seq read density on three groups of genes with different levels of PI, EI, and TI quantified from KAS-seq data, and found that quantification from two independent assays is highly consistent (Fig. 3d–i). These findings indicate that the transcription initiation and termination processes of different genes are dynamically regulated (Fig. 3j). Collectively, PI, EI, and TI provide a quantitative summary of the transcriptionally engaged RNA Pol II activities at three different stages of the transcription cycle.

### 3.4 The identification of SST enhancers

Transcribed enhancers are short regulatory elements that synthesize enhancer RNA (eRNA) by proximal-paused RNA Pol II (De Santa *et al*. 2010), which are generally associated with specific transcription factor binding and experience more long-range interactions than regular enhancers (Hirabayashi *et al*. 2019; Wu *et al*. 2020). Numerous studies have reported that transcribed enhancers are more likely to be functional than those identified using only histone marks or chromatin accessibility data. In previous studies, we defined transcribed enhancers using ssDNA signals by directly overlapping KAS-seq peaks with enhancers indicated by distal H3K27ac or DNase I hypersensitive sites (DHSs), termed as SST enhancers. However, this method may produce false positives, since it cannot distinguish KAS-seq peaks mediated by proximal-paused RNA Pol II from those mediated by transcription elongation, both of which can display ssDNA signals on distal H3K27ac peaks and DHSs. To address this issue, KAS-Analyzer introduces a new filtering strategy to select enhancers with proximal-paused RNA Pols by comparing all KAS-seq peaks to nearby regions. KAS-seq peaks mediated by transcription elongation will not be significantly different from the nearby regions and can be eliminated.

**Figure 4.**
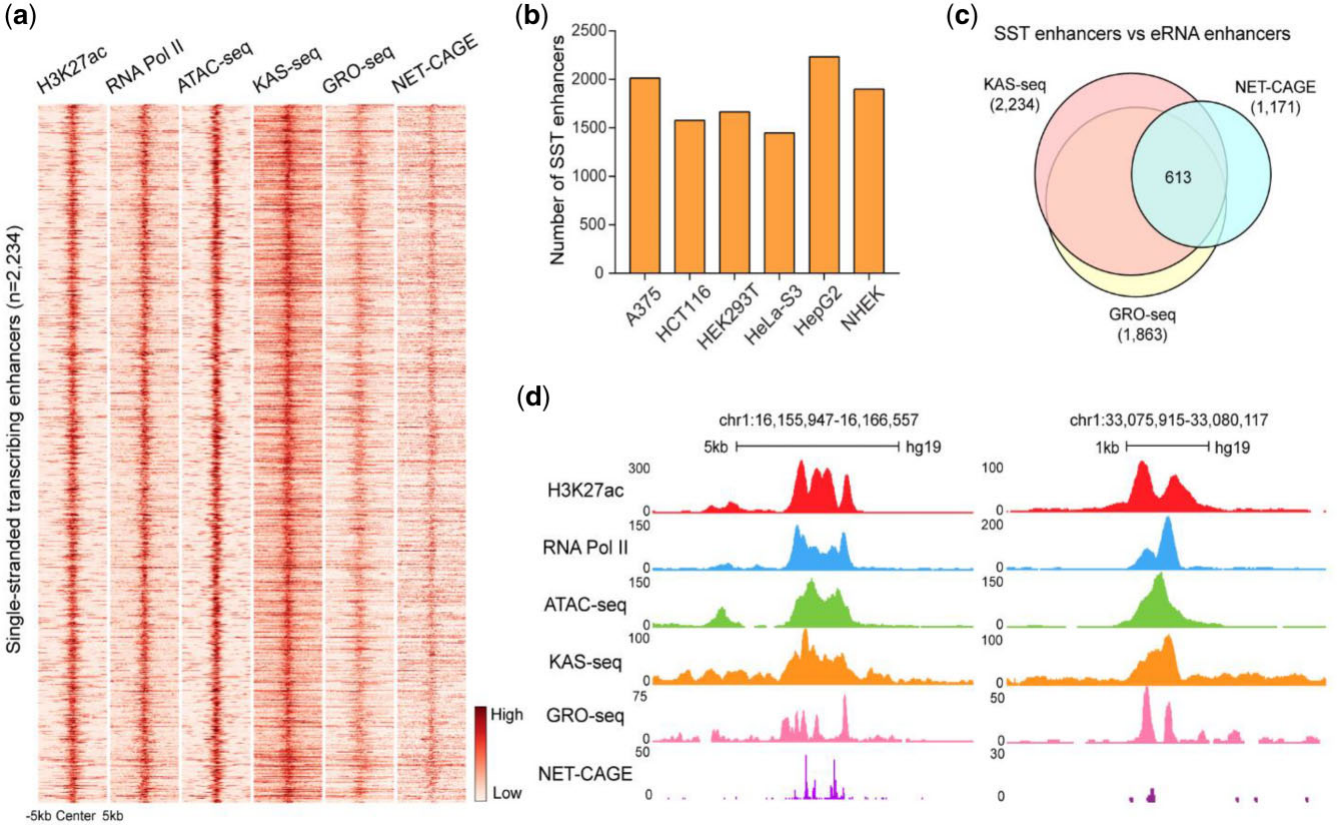
Identification of SST enhancers using KAS-seq data. (a) Heatmap plot showing the H3K27ac ChIP-seq, RNA Pol II ChIP-seq, ATAC-seq, KAS-seq, GRO-seq, and NET-CAGE read density in 2234 SST enhancers identified using KAS-seq data from HepG2 cells. Active enhancers were defined as distal H3K27ac peaks identified using ChIP-seq data. (b) Bar plot showing the number of SST enhancers identified in the example KAS-seq dataset generated from six human cell lines: A375 (2013); HCT116 (1578); HEK293T (1665); HeLa-S3 (1448); HepG2 (2234); and NHEK (1900). (c) Venn diagram showing the overlap between transcribed enhancers defined by KAS-seq, GRO-seq, and NET-CAGE data in HepG2 cells. (d) Snapshots of custom tracks in UCSC genome browser showing the pattern of H3K27ac, RNA Pol II, ATAC-seq, KAS-seq, GRO-seq, and NET-CAGE read density on two SST enhancer examples identified using KAS-seq data from HepG2 cells.

For each enhancer overlapping a KAS-seq peak, we first delineate two regions of equal size, referred to as “enhancer shores,” adjacent to the upstream and downstream boundaries of the enhancer. Next, we compute the ssDNA read density within the enhancer and its corresponding shores. Enhancers exhibiting a significantly higher ssDNA density (1.5-fold enrichment; one-way ANOVA with a *P*-value ≤ .05) in comparison to their enhancer shores are identified as SST enhancers. To illustrate this functionality, we applied KAS-Analyzer to the example KAS-seq datasets generated in six human cell lines. As a result, we identified 2234 SST enhancers from 26 719 active enhancers (distal H3K27ac peaks) in HepG2 cells, which strongly overlapped with open chromatin regions defined by ATAC-seq (90.8%, 2029/2234) and RNA Pol II binding (85.9%, 1919/2234) (Fig. 4a). However, we also identified 5102 enhancers that overlap with KAS-seq peaks but did not meet the filtering criteria for displaying significantly higher ssDNA density; these are referred to as non-SST enhancers (Fig. 4b). Our results are also supported by the results from two nascent RNA-based orthogonal assays, GRO-seq and NET-CAGE. We found that 84.6% (1889/2234) of SST enhancers have detectable eRNA transcription according to GRO-seq and NET-CAGE data (Fig. 4c and d). In contrast, only 15.5% (791/5102) of non-SST enhancers demonstrated detectable eRNA transcription according to these same datasets, which illustrates the benefit of our filtering strategy in eliminating false positives compared with the direct overlapping of KAS-seq peaks with enhancer regions (Fig. 4b and f). Moreover, we identified more transcribed enhancers in KAS-seq (2234) data than GRO-seq (1863) and NET-CAGE (1171) data, which is expected since nascent eRNA transcripts are readily degraded by the nuclear exosome complex soon after their synthesis (Kilchert *et al.* 2016), whereas ssDNA is stable (Fig. 4e and f).

### 3.5 Genome-wide R-loop identification using spKAS-seq data

**Figure 5.**
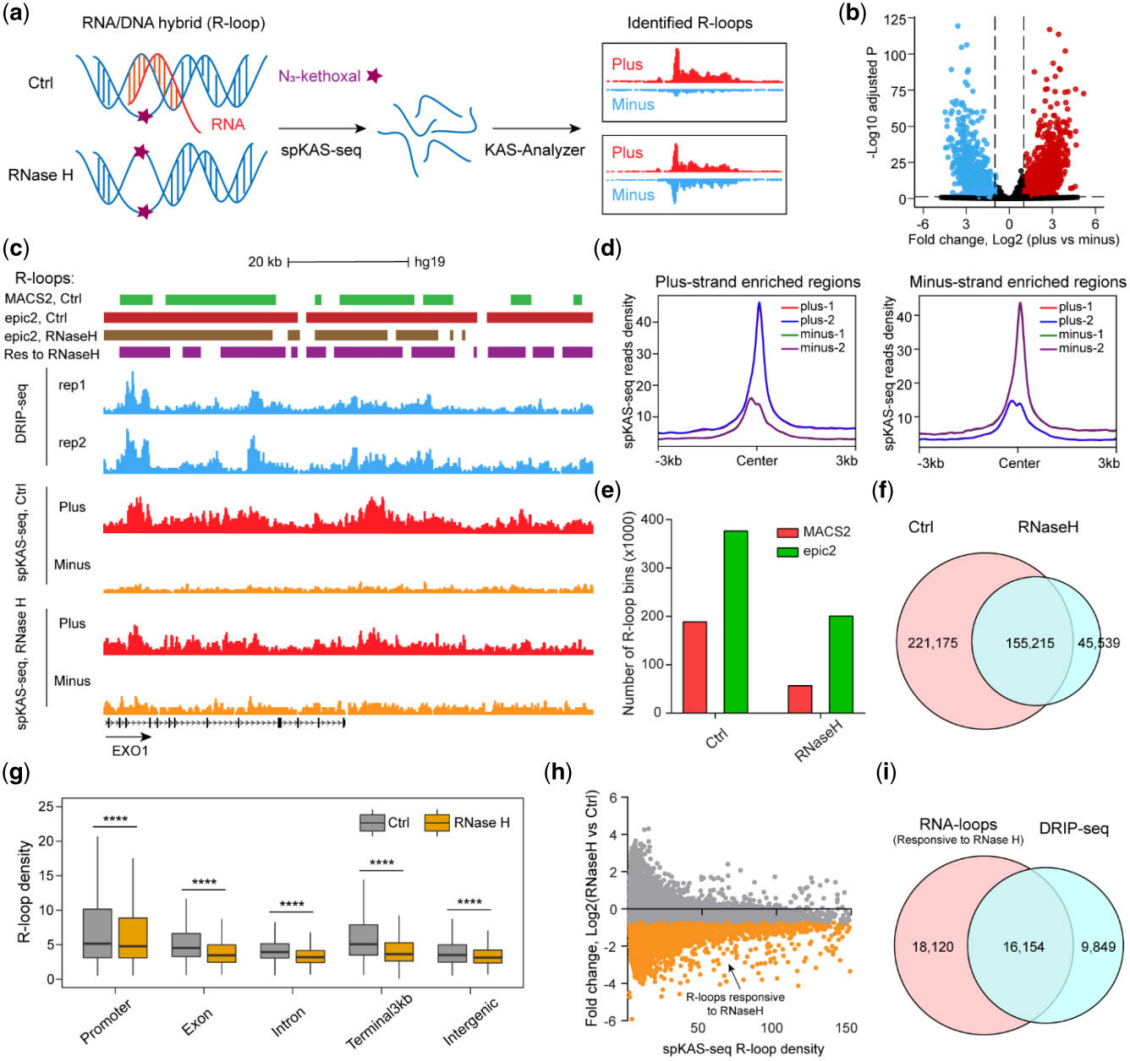
Identification of RNase H-responsive R-loops using spKAS-seq data. (a) Schematic diagram of KAS-Analyzer using spKAS-seq to map the genome-wide R-loop structures sensitive to RNase H treatment. (b) Volcano plot showing the number of sliding windows with unbalanced spKAS-seq read numbers mapped on plus or minus strands in HEK293T cells. Red dots indicate the sliding windows with a significantly higher spKAS-seq reads number on the plus strand (*n* = 192 056). Blue dots indicate the sliding windows with a significantly higher spKAS-seq reads number on the minus strand (*n* = 184 334). (c) Snapshots of spKAS-seq data from UCSC genome browser showing the pattern of unbalanced spKAS-seq reads mapped to plus (red) or minus (orange) strand on one example region identified by KAS-Analyzer using spKAS-seq data generated in HEK293T cells. Snapshots of DRIP-seq data (blue) from the UCSC genome browser for the same regions are provided for comparison. R-loops identified using the sliding windows overlapped with KAS-seq peaks by various tools are labeled, as well as R-loops (based on epic2-identified peaks) responsive to RNase H treatment (Res to RNase H). (d) Metagene profile plots showing the spKAS-seq read density with strand specificity on the identified promoter R-loops enriched on plus and minus strands. (e) Bar plot showing the number of sliding windows (R-loop bins) with significantly unbalanced spKAS-seq reads mapped to the plus or minus strand in both control and RNase H-treated HEK293T cells. (f) Venn diagram showing the overlap of R-loop bins identified using spKAS-seq data in both control and RNase H-treated HEK293T cells. (g) Boxplot showing the comparison of the density of R-loops within R-loop bins across various genomic features in both control and RNase H-treated HEK293T cells. *P*-values were calculated using two-sided paired Student’s *t*-test. *P* < .0001 was shown as ****. (h) MAplot illustrating the number of R-loop bins that are responsive to RNase H treatment in HEK293T cells. (i) Venn diagram showing the overlap between RNase H-responsive R-loops and DRIP-seq defined R-loops in HEK293T cells.

R-loops are three-stranded structures that consist of an RNA–DNA hybrid and a displaced single-stranded DNA (ssDNA) (Aguilera and Garc**í**a-Muse 2012, Sanz *et al.* 2016). Genome-wide R-loop detection has primarily relied on technologies that enrich for RNA–DNA hybrids using the S9.6 monoclonal antibody or catalytically inactive RNase H before performing high-throughput sequencing, such as DRIP-seq, R-ChIP, and mapR (Phillips *et al.* 2013, Chen *et al.* 2017, 2019, Sanz and Chédin 2019, Yan *et al.* 2019). We have developed spKAS-seq, a strand-specific variant of KAS-seq that uses asymmetric ssDNA exposure on the two strands to infer R-loops (Fig. 5a) (Wu *et al.* 2022). Furthermore, by comparing spKAS-seq data from wide-type and RNase H-treated cells, we can identify RNase H-responsive R-loops, which helps to minimize false positives and background noise (Fig. 5a). We thus applied KAS-Analyzer to the spKAS-seq data generated in HEK293T cells for the identification of windows with significantly unbalanced ssDNA density. First, KAS-Analyzer separates the uniquely mapped reads according to their strand information. Strand-specific reads are then employed to construct a read-count matrix for each 500 bp windows that interacts with spKAS-seq peaks. Finally, we performed differential analysis using DESeq2 to obtain a catalog of R-loops. By utilizing spKAS-seq data in HEK293T from two biological replicates, we identified 192 056 and 184 334 windows with significant more uniquely mapped reads on the positive and negative strands, respectively (Fig. 5b–d).

To verify that the imbalances of ssDNA on two strands are indeed R-loop positives, we applied KAS-Analyzer to analyze spKAS-seq data in HEK293T cells treated with RNase H, the gold standard approach for R-loop detection (Wu *et al.* 2022). Our findings demonstrate that regions with asymmetric ssDNA signals were notably responsive to RNase H treatment, which substantially reduces the number of windows with unbalanced ssDNA density, regardless of genomic distribution (Fig. 5e–g). These results suggest that spKAS-seq offers a powerful approach for R-loops identification. Next, we compared our results to R-loops identified with DRIP-seq, a method that utilizes the S9.6 monoclonal antibody to capture R-loops. We found that more than half of the R-loops identified by DRIP-seq (62.1%, 16 154/26 003) overlapped with RNase H-responsive R-loops detected using spKAS-seq in HEK293T cells (Fig. 5h and i). This discrepancy could be due to methodological or technical differences; for example, DRIP-seq may inadvertently perturb R-loops and can be biased toward certain chromatin regions.

### 3.6 Differential RNA polymerases activity analysis for time-course KAS-seq data

**Figure 6.**
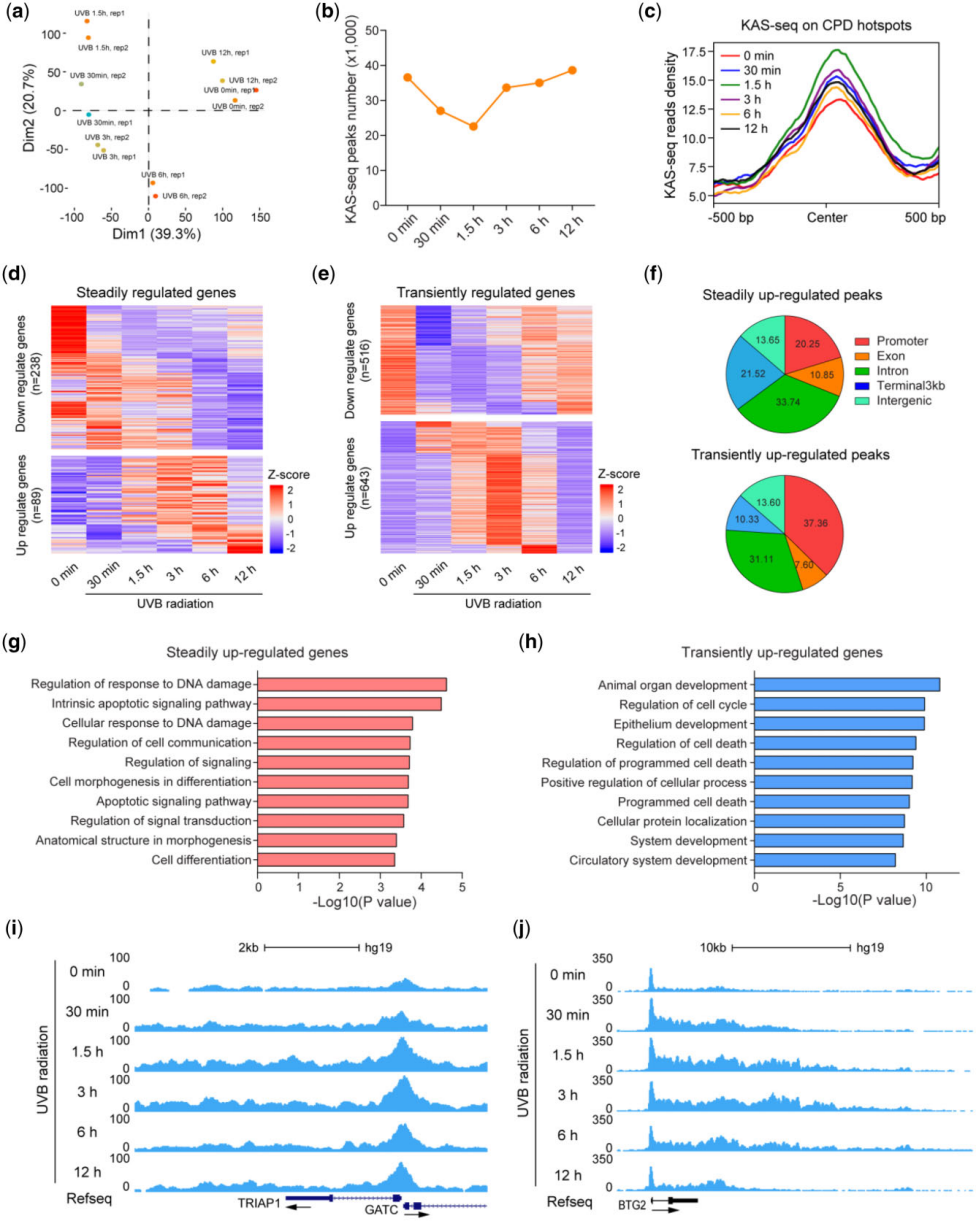
Differential RNA Pol II activity analysis for time-course KAS-seq data in NHEK cells. (a) Principal component analysis (PCA) plot of KAS-seq data generated in NHEK cells treated with UVB radiation for 0 min (sham irradiation), 30 min, 1.5 h, 3 h, 6 h, and 12 h for two replicates (rep1 and rep2). (b) Number of KAS-seq peaks called using KAS-seq data in NHEK cells treated with UVB radiation at different time points. (c) Metagene profile plots showing the KAS-seq read density on UV-induced CPDs hotspots. (d and e) Heatmap plots showing the dynamics of global RNA Pol II activity patterns of steadily regulated genes (d) and transiently regulated genes (e) based on normalized read counts of time-course KAS-seq data. (f) Pie charts showing the distribution of bins (2 kb) with steadily and transiently up-regulated KAS-seq signals on different genomic features in NHEK cells in response to UVB exposure. (g and h) Bar plots showing the GO analysis for steadily up-regulated genes (g) and transiently up-regulated genes (h). (i and j) Snapshots of time-course KAS-seq data custom tracks from UCSC genome browser showing the KAS-seq read density on example steadily (TRIAP1) (i) and transiently (BTG2) (j) up-regulated genes in NHEK cells treated with UVB radiation at different time points.

Time-course KAS-seq experiments can provide insights into the regulatory mechanisms of gene expression by capturing transient transcriptional dynamics with temporal resolution (Wu *et al.* 2020, Lyu *et al.* 2022). KAS-Analyzer supports differential RNA Pols activity analysis for time-course KAS-seq data. As an example, we applied KAS-Analyzer to a time-course KAS-seq dataset with human epidermal keratinocyte (NHEK) cells treated with UVB radiation for 0** **min (sham irradiation), 30** **min, 1.5 h, 3 h, 6 h, and 12 h, respectively. We observed that UVB radiation treatment clearly induced transcriptional changes from 30** **min to 12 h, leading to distinct KAS-seq profiles at each time point (Fig. 6a). Exposure to UVB radiation typically leads to the formation of photoproducts, such as cyclobutane pyrimidine dimers (CPDs), which can induce DNA damage and subsequently impact cellular functions (Drouin and Therrien 1997). Nevertheless, the formation of these photoproducts can also stimulate DNA repair mechanisms. We observed that the number of KAS-seq peaks started to decline at 30 min of UVB radiation, reached its lowest point at 1.5 h, and then began to rise at 3 h and beyond (Fig. 6b). Interestingly, we found that KAS-seq signals exhibited an inverse enrichment pattern on CPD hotspots (Jiang *et al.* 2021), with the highest enrichment observed at 1.5 h of UVB radiation exposure (Fig. 6c). This finding is consistent with the fact that ssDNA is frequently present at sites of DNA damage during the DNA repair process.

From differential time-course RNA Pols activity analysis, we identified 327 genes (89 up and 238 down) as “steadily regulated genes” with continuous effects (Fig. 6d), and 1161 genes (643 up and 516 down) as “transiently regulated genes” with fleeting effects (Fig. 6e). In addition, we found that the steadily and transiently up-regulated KAS-seq signals primarily occur within the gene bodies, with less frequent occurrence in promoters, terminators (3 kb), and intergenic regions (Fig. 6f). Moreover, gene ontology (GO) analysis revealed that steadily up-regulated genes were mainly related to DNA damage and apoptosis signaling pathways (Fig. 6g), and transiently up-regulated genes were significantly enriched in critical biological processes, including cell development, cell cycle, and cell death (Fig. 6h). For instance, *TRIAP1* was identified as a steadily up-regulated gene that prevents apoptosis by mediating intramitochondrial transport of phosphatidic acid (Potting *et al.* 2013), whereas *BTG2*, which was transiently up-regulated with UVB radiation treatment only for 30 min to 6 h (Fig. 6i and j), is involved in cell cycle processes and functions as an antiproliferative p53-dependent component of the DNA damage cellular response pathway (Rouault *et al.* 1996). The in-depth analysis conducted using KAS-Analyzer on the time-course KAS-seq dataset offered valuable insights into the cellular response and DNA repair processes that occur following UVB radiation exposure in NHEK cells.

## 4 Discussion

In this study, we have presented KAS-Analyzer, a user-friendly, versatile, one-stop-shop methodological framework suitable for diverse applications using KAS-seq or spKAS-seq. Unlike the previous pipeline (KAS-pipe), which only includes a few stand-alone shell scripts (Lyu *et al.* 2022), KAS-Analyzer adopts a new framework to implement functionalities under the usage: KAS-Analyzer <subcommand> [options]. This framework is convenient to update with new features and has been adopted in many other popular packages, such as *deeptools* and *samtools* (Li *et al.* 2009, Ram**í**rez *et al.* 2014).

It is crucial to note that the quality control metrics and thresholds employed in this study to evaluate KAS-seq data quality are primarily recommended for live human and mouse cell lines. These criteria may not be ideal for frozen tissues or samples derived from species with smaller genomes. KAS-Analyzer provides a robust methodological framework to study the transient dynamics of each step in the transcription cycle. Specifically, it includes several novel metrics to capture the activity of transcriptionally engaged RNA Pol II, such as PI, EI, and TI. Our work also revealed new insight into the mechanisms responsible for transcription termination during elongation. Currently, two models have been proposed for the transcription termination process: either (1) RNA Pol II is released following an allosteric change in the elongation complex (the allosteric model) or (2) the elongation complex is dismantled following degradation of the nascent RNA transcripts by a 5'–3' exoribonuclease (the torpedo model). Additionally, N3-kethoxal in KAS-seq can rapidly (within 5 min) and sensitively react with guanine bases in ssDNA. Despite this, we did not observe any significant bias toward DNA sequence composition or GC content in our experiments, we do not expect variation in GC content to affect the accuracy of the estimated transcription-related metrics in most cases (Supplementary Fig. S2a–c). However, it may still be crucial to consider potential biases toward GC-rich regions when applying KAS-seq in specific contexts.

One limitation of our current workflow pertains to the identification of genome-wide R-loops. Canonical R-loops form when RNA invades the DNA duplex and anneals to its template DNA. Yet, non-canonical DNA structures other than R-loops, such as triple-strand DNA (H-DNA), may also asymmetrically expose ssDNA, thereby contributing to a small portion of defined R-loop signals. However, the definitive approach used to distinguish between true R-loops and false positives noise is to treat spKAS-seq samples with RNase H, which is a non-sequence-specific endonuclease enzyme that catalyzes the cleavage of RNA in R-loop structures. Therefore, we have incorporated a “RNaseH” function in KAS-Analyzer that enables the identification of canonical R-loops that are sensitive to RNase H treatment (RNase H-responsive R-loops) by comparing spKAS-seq data from wild-type and RNase H-treated cells.

Lastly, in our established protocol, KAS-Analyzer utilizes RPKM for initial normalization in time-course differential KAS-seq analysis. While this approach is efficient for normalization in response to variations in library size, it may not optimally address potential interferences brought on by significant changes in global signal. We acknowledge that incorporating spike-in controls, such as exogenous ssDNA oligos, could provide a more effective means of normalizing global KAS-seq signal fluctuations. Such controls present an opportunity to mitigate the impact of varying global signal levels on the analysis, thereby increasing the reliability and robustness of the results obtained.

## 5 Conclusion

KAS-Analyzer is a novel, comprehensive computational framework that enables researchers to study the dynamic activities of transcriptionally engaged RNA Pols and transient transcriptional regulatory programs. Using the example dataset consisting of KAS-seq data generated from six human cell lines, we showcase novel features implemented in KAS-Analyzer and diverse applications of the KAS-seq technology, including calculation of transcription-related metrics, improved identification of SST enhancers, high resolution mapping of R-loops, and differential RNA Pols activity analysis. KAS-Analyzer provides a powerful workflow for pre-processing, analysis, and interpretation of KAS-seq and spKAS-seq data. We plan to enhance future versions of KAS-Analyzer by developing more analytical tools for new applications of KAS-seq experiments.

## Supplementary Material

vbad121_Supplementary_Data

## Data Availability

Raw sequencing data of KAS-seq experiments in A375, HCT116, HepG2, HeLa-S3, and NHEK cells have been deposited at Gene Expression Omnibus (GEO): GSE202044. KAS-seq data from HEK293T cells used in this study can be downloaded from GEO: GSE139420. spKAS-seq data from wide-type and RNase H treated HEK293T cells can be downloaded from GEO: GSE192822. KAS-Analyzer is open source under the GPL-3.0 license. KAS-Analyzer is available at https://github.com/Ruitulyu/KAS-Analyzer. A copy of the source code has also been deposited in Zenodo (DOI: https://doi.org/10.5281/zenodo.6519166).
