# Supplementary material for: KAS-Analyzer: a novel computational framework for exploring KAS-seq data
Source: Bioinform Adv. 2023 Sep 8;3(1):vbad121. doi: 10.1093/bioadv/vbad121 (PMC10516523; doi:10.1093/bioadv/vbad121)
Supplement: vbad121_Supplementary_Data [file vbad121_supplementary_data.pdf]

Supplementary Materials for

KAS-Analyzer: a novel comprehensive framework for exploring KAS-seq data

Ruitu Lyu[1,2], Tong, Wu[1,2], Gayoung Park[4], Yu-Ying He[4], Mengjie Chen[4,5*], Chuan He[1,2,3*]

[1]Department of Chemistry, University of Chicago, IL, USA.

[2]Howard Hughes Medical Institute, University of Chicago, IL, USA.

[3]Department of Biochemistry and Molecular Biology, Institute for Biophysical Dynamics, University of Chicago, IL, USA.

[4]Department of Medicine, The University of Chicago, Chicago, IL, USA

[5]Department of Human Genetics, The University of Chicago, Chicago, IL, USA

*Corresponding author. Email: mengjiechen@uchicago.edu, chuanhe@uchicago.edu,

This PDF file includes:

Fig. S1 & S2

a

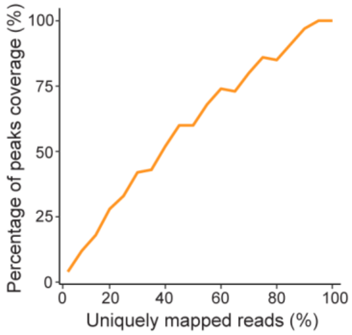

b

Library complexity metrics of KAS-seq experiments

Samples	PBC	Bottlenecking	NRF	Complexity
A375	0.81	None	0.78	Ideal
HCT116	0.81	None	0.79	Ideal
HEK293T	0.88	None	0.83	Ideal
HeLa-S3	0.58	Moderate	0.54	Acceptable
HepG2	0.8	None	0.75	Ideal
NEHK	0.79	None	0.77	Ideal

Figure S1. Quality control of KAS-seq data. a, Line plot showing the saturation analysis pf KAS-seq data in HCT116 cells. b, Table chart showing the library complexity metrics calculated using example KAS-seq datasets in 6 human cell lines.

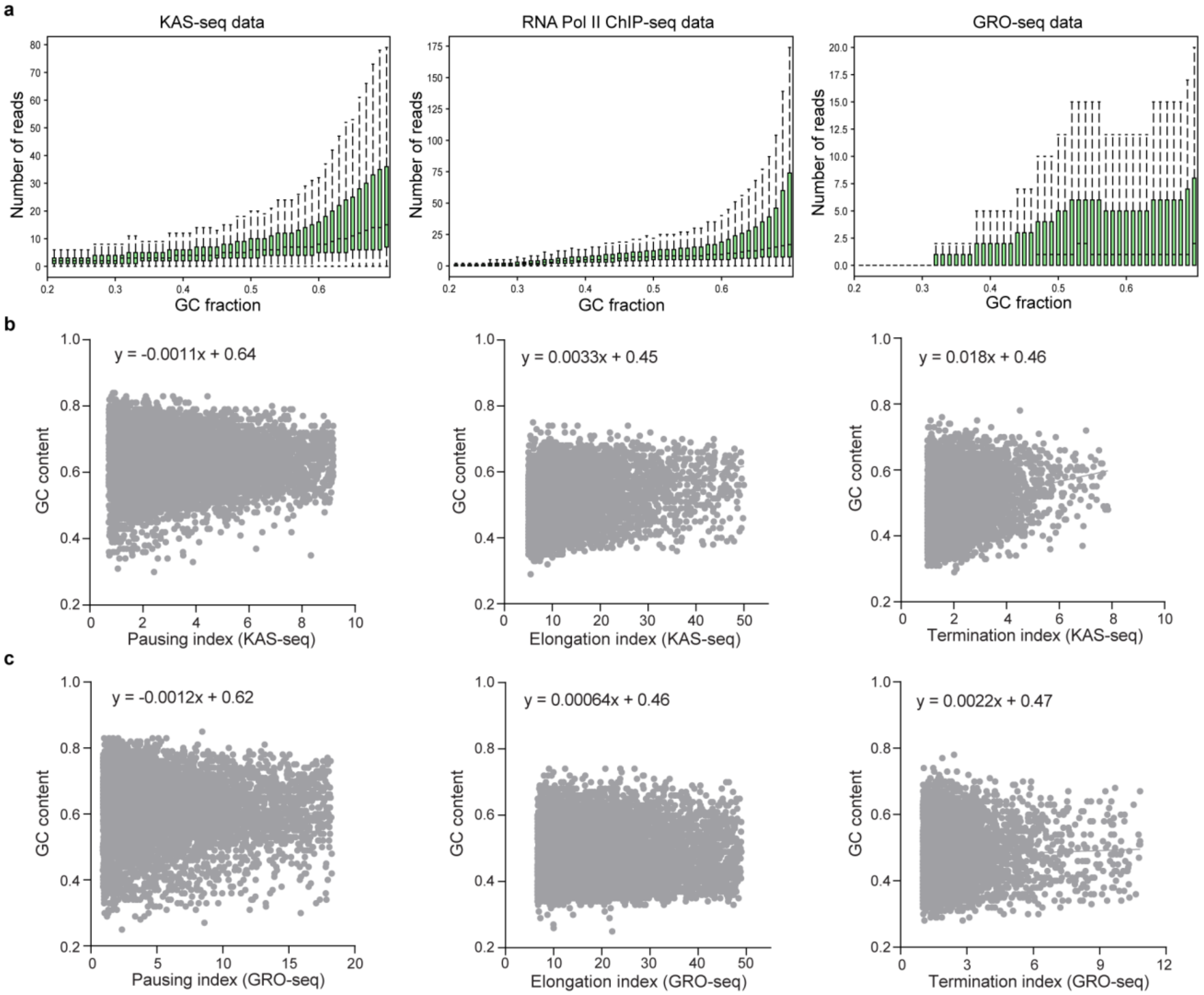

Figure S2. Correlation between GC content and indexes estimated using KAS-seq and GRO-seq data.
a, The read distribution of KAS-seq (left), Pol II ChIP-seq (middle), and GRO-seq (right) signals respect to different GC fractions. b, Scatterplots showing the correlation between GC content and pausing index (left), elongation index (middle), and termination index (right) estimated using KAS-seq data in HCT116 cells. The equation shown represents the GC content (y) as a linear function of index (x). c, Scatterplots showing the correlation between GC content and pausing index (left), elongation index (middle), and termination index (right) estimated using GRO-seq data in HCT116 cells. The equation shown represents the GC content (y) as a linear function of index (x).
